# Genetic analysis of pigment production in the fungus *Exophiala dermatitidis* mutant strains obtained via nontargeted UV mutagenesis

**DOI:** 10.1093/g3journal/jkaf205

**Published:** 2025-09-03

**Authors:** Kamaldeep Chhoker, Georg Hausner, Steven D Harris

**Affiliations:** Department of Biological Sciences, University of Manitoba, Winnipeg, MB, Canada R3T 2N2; Department of Microbiology, University of Manitoba, Winnipeg, MB, Canada R3T 2N2; Department of Biological Sciences, University of Manitoba, Winnipeg, MB, Canada R3T 2N2; Department of Plant Pathology, Entomology and Microbiology, Iowa State University, Ames, IA 50011, United States

**Keywords:** *Exophiala dermatitidis*, 1-8, DHN melanin, PKS1, hyperpigmented, albino, phenotypic assay, single nucleotide variants

## Abstract

*Exophiala dermatitidis* is a polyextremotolerant black yeast species. *E. dermatitidis* produces 1,8-dihydroxynaphthalene (DHN) melanin via the polyketide synthase 1 (*PKS1*) pathway enabling it to survive harmful conditions. This study focused on random nontargeted mutagenesis to obtain albino (*alb*) and hyperpigmented (*hyp*) mutants. Notably, all 17 *alb* mutants possessed mutations in *PKS1* whereas the 113 hyperpigmented (*hyp*) mutants harbored mutations impacting a range of functions. Cell morphology and phenotypic assays showed additional differences between the *alb* and *hyp* mutants. Strikingly, 3 of the albino mutants (*alb1*, *alb2*, and *alb3*) were conditional in that despite the presence of mutations in *PKS1* they were able to produce melanin upon exposure to different carbon sources. These mutants otherwise shared similar cell morphology and growth patterns with the obligate albinos. No additional shared mutations were found among the conditional albinos. Temperature and UV irradiation assays demonstrated reduced growth of albino mutants at higher temperatures (i.e. 42 °C) and a greater sensitivity to higher doses of UV. Single nucleotide variant calling showed that some hyperpigmented mutants had a greater number of single nucleotide variants compared to albino strains. To date, this is the first study to generate and characterize conditional albino mutants in *E. dermatitidis* with the ability to recover melanin production.

## Introduction

Black yeasts, which are sometimes referred to as dematiaceous fungi, are a group of fungi that have black-brown appearance due to the association of melanin with their cell walls (Kejžar et al. 2013; Selbmann et al. 2015). Many species of black yeasts have been found growing in toxic niches, nutrient poor conditions and manmade environments (Onofri et al. 2008; Gostincar et al. 2012; Coleine et al. 2022; Gostinčar and Gunde-Cimerman 2023; Medina-Armijo et al. 2024). Another key aspect of black yeasts is morphological plasticity which refers to their ability to switch morphology thereby enabling some of them to be opportunistic pathogens of both warm- and cold-blooded vertebrates (Slepecky and Starmer 2009; de Hoog 2014). Black yeasts can shift from single-celled yeasts to multicellular filamentous forms (Butin et al. 1996; Butler and Day 1998). This switch in their growth forms increases the surface/volume ratio of the fungi allowing them to absorb and transport more nutrients through the cell membrane when growing in nutrient poor habitats (Sterflinger and Krumbein 1995; Zalar et al. 1999). *Exophiala dermatitidis* (also referred to as *Wangiella dermatitidis*; see Chen et al. 2014) is one such opportunistic black yeast found growing in soil, toxic and arid niches as well as in nutrient poor manmade environments ranging from steam baths, railway ties and dishwashers (Hamada and Abe 2009; Döğen et al. 2013; Zupančič et al. 2016). Emergence of *E. dermatitidis* as an opportunistic pathogen has been reported in immunocompromised patients where it is able to cause phaeohyphomycosis, chromoblastomycosis, and fatal infections of central nervous system. Notably, the ability of *E. dermatitidis* to switch its morphology from yeast-like to a multicellular hyphal form at 37 °C enables it to survive and adapt to the mammalian host (de Hoog 2014; Olsowski et al. 2018; Vasquez et al. 2018; Wang et al. 2019; Beniwal et al. 2023).

In general, melanization of the cell wall and the ability of some black yeasts to switch their growth form are 2 key adaptations that allow them to cope with stressful environmental conditions (Gorbushina 2007). Melanin is typically found in the outer regions of the cell wall in most species but in some cases can be found aggregated to the cell wall surface (Bayry et al. 2014). Fungal melanin provides various benefits to black yeasts such as protection against oxidative stress (Cordero and Casadevall 2017), radiation (Dadachova et al. 2007), UV, and hyperosmotic stress (Wollenzien et al. 1995; Kogej et al. 2007).

*E. dermatitidis* produces 1,8-dihydroxynaphthalene (DHN) melanin, which has been shown to provide protection from oxidative stress, enzymatic lysis and phagocytosis during infection (Pihet et al. 2009; Kurbessoian et al. 2023). The starting precursors for the synthesis of 1,8-DHN melanin can either be acetyl-CoA or malonyl-CoA which are produced endogenously (Nosanchuk et al. 2015). Besides 1,8-DHN melanin, genome annotation has revealed that *E. dermatitidis* also possesses the capacity to produce L-dihydroxyphenylalanine (L-DOPA) melanin and pyomelanin via the L-tyrosine degradation pathway (Chen et al. 2014; Poyntner et al. 2018; Carr et al. 2023). In brief, the biosynthesis of DOPA melanin begins with tyrosine, which is oxidized by oxygen followed by tyrosinase that forms levodopa and then dopaquinone (Simon and Peles 2010; Cao et al. 2021). In the DOPA melanin pathway, hydroxylation of L-tyrosine to dopaquinone or the oxidation of L-DOPA to dopaquinone is catalyzed by tyrosinase or laccase, respectively (Pomerantz and Warner 1967). Pyomelanins are derived from the oxidative polymerization of nitrogen-free precursors such as homogentisic acid (HGA) (Funa et al. 2005; Seo and Choi 2020). Pyomelanin originates from the catabolism of either tyrosine or phenylalanine. Nevertheless, the specific roles of these alternate types of melanin relative to that of 1,8-DHN melanin remain unknown. Notably, targeted mutagenesis of the *E*. *dermatitidis PKS1* gene is sufficient to generate albino mutants (Feng et al. 2001; Poyntner et al. 2018; Malo et al. 2021). This implies that the other melanin pathways might not provide any contribution to melanin production. Alternatively, the targeted nature of the prior mutagenic approaches might simply have precluded the identification of mutations affecting the alternate pathways. Accordingly, the objective of this study was to combine conventional random mutagenesis with whole genome sequencing to determine the spectrum of mutations that underlie both albino (*alb*) and hyperpigmented (*hyp*) phenotypes. Besides identifying genes that in addition to *PKS1* can trigger an albino phenotype when mutated, the screen was also expected to provide insight into the regulatory mechanism that control melanin production in *E*. *dermatitidis*.

## Materials and methods

### Media and strains

Media used in this study include yeast extract peptone dextrose (YPD): (10 g yeast extract, 20 g peptone, 20 g dextrose, and 20 g agar per 1 L), yeast extract peptone galactose (YPG): (10 g yeast extract, 20 g peptone, 20 g galactose, and 20 g agar per 1 L), malt extract agar (MEA): (20 g malt extract, 2 g peptone, 10 g dextrose, and 20 g agar per 1 L), and minimal media (MN): (10 g dextrose, 50 ml 20 × nitrate salts* and 1 ml Hutner's TE** per 1 L); *20 × nitrate salts (1 L: 120 g NaNO_3_, 104 g KCL, 10.4 g MGSO_4_-7H_2_O, 30.4 g KH_2_PO_4_; **Hutner's TE (100 ml: 2.2 g ZNSO_4_-7H_2_0, 1.1 g H_2_BO_3_, 0.5 g MnCl_2_-4H_2_0, 0.5 g FeSO_4_-7H_2_O, 0.17 g CoCl_2_-6H_2_O, 0.16 g CuSO_4_-5H_2_O, 0.15 g Na2MoO_4_-2H_2_O, 5 g EDTA (disodium salt).

The *E*. *dermatitidis* reference strain UT8656 (=ATCC 34100, =*Exophiala dermatitidis* CBS 525.76) was treated as the wildtype strain as described in (Chen et al. 2014). This strain was used for mutagenesis to obtain pigmentation mutant strains. For long-term storage, the wildtype strain and the mutant strains (*alb* and *hyp*) obtained via UV mutagenesis were kept in 30% glycerol at −80 °C.

### UV mutagenesis

An unbiased forward genetic approach was employed to screen for *E*. *dermatitidis* mutants with altered pigmentation phenotypes ranging from complete loss of pigment to hyperpigmentation. Briefly, wildtype strain UT8656 was initially plated on YPD (Supplementary Fig. 1). Four to 5 discrete colonies were isolated from the plates and combined into 1 ml microcentrifuge tubes (Fisher Scientific, Nepean, ON, Canada) containing autoclaved sterile distilled water. Diluted 250 µl mixtures were plated onto YPD and subjected to UV mutagenesis (UVB) using a Stratagene Stratalinker UV1800 crosslinker (Marshall Scientific, Hampton, NH, United States) at setting energy 1,000 × 100 µW/cm^2^. The plates were removed, immediately wrapped in tinfoil to limit photoreactivation, and placed in an incubator at 28 °C. After 2 d, the plates were unwrapped and returned to the incubator. Growth was monitored and mutant colonies started to appear after about 7 d at 28 °C. After 7 to 10 d, colonies were examined for color and morphology. The *E. dermatitidis* wildtype strain appears brown on YPD so any colonies that appeared white (*alb*) or black (*hyp*) were selected. One hundred YPD plates were subjected to random mutagenesis to obtain *E. dermatitidis* mutant strains. Mutant colonies that appeared fuzzy or crusty were also picked. The main difference between fuzzy and crusty was attributed to the presence of aerial hyphae in the fuzzy mutants whereas crusty mutants lacked aerial hyphae. Fuzzy mutants were easier to scrape off from the medium whereas crusty mutants tend to be invasive and embedded in the medium. Mutant isolates were sub-cultured on YPD plates to verify the selected phenotype and to generate material for subsequent phenotypic testing.

### Study of cell morphology

**Fig. 1.**
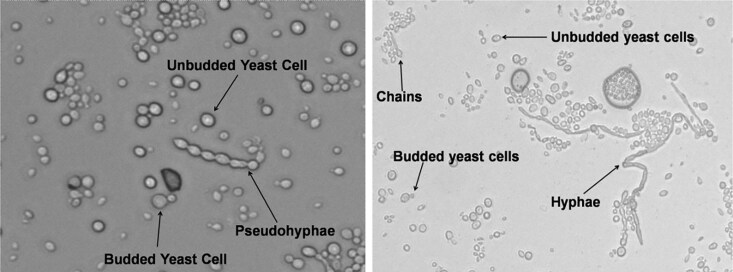
Different cell types observed in *E. dermatitidis* mutant strains (unbudded yeast cells, budded yeast cells, chains, pseudohyphae, and hyphae). Supplementary Fig. 5 represents additional examples of images obtained using an EVOS M5000 desktop microscope.

To observe morphological phenotypes, wildtype and mutant isolates stocks were cultured onto YPD plates. Once the strains had grown, single colonies were picked and mounted in sterile water on glass slides for observation using an EVOS M5000 (Fischer Scientific) desktop microscope. Cell imaging software (FL Auto 2 Cell Imaging software) was used according to the manufacturer's guidelines to count the following cell types: unbudded yeast cells, budded yeast cells, chains, pseudohyphae, and hyphae (Fig. 1). Manual counting was performed because small cell sizes resulted in discrepancies in the automated cell counts. For each strain, 3 replicates were examined, and photographs of the slides were taken using the built-in camera of EVOS M5000 (Supplementary Fig. 5). Two hundred cells were counted from each photograph and the final cell count was based on the average number of different cells observed in the 3 replicates.

### UV resistance and temperature assays

Mutants were subjected to different phenotypic assays including UV tolerance as well as responses to different growth temperatures and media types. Briefly, *E. dermatitidis* mutants were plated onto YPD and left to grow at 28 °C. Isolated colonies were suspended in sterile distilled water and diluted 10-fold. A semi-quantitative spot dilution method was used to observe the growth of the mutants in each assay (Supplementary Fig. 2). For the temperature assay, 4 different temperatures were initially selected: 4, 10, 28, and 42 °C. No visible growth was observed at 4 °C even after 3 mo so only the other 3 temperature assays were recorded. For UV resistance, spot plates were subjected to 6 different treatments: no UV (control), 500, 750, 1,000, 1,250, and 1,500 energy 1,000 × 100 µW/cm^2^. Growth was recorded after 2 wk of incubation and again after an additional 3 wk. Growth was scored on a scale of 1 to 7 where 1 indicated complete inhibition/no growth and 7 indicated no inhibition (Supplementary Fig. 3).

### Carbon source utilization

ID 32 C-strips (Mediray) were used to test the effect of different carbon sources on the growth and pigmentation of each mutant. For this analysis, wildtype and all 17 albino mutants were plated onto YPD and inoculated on the ID 32 C-strips following the manufacturer's protocol. Briefly, for each sample, several discrete colonies were picked and added to the provided ampule of API Suspension Medium (2 ml). 250 µl of the suspension from the ampule AP1 was then added to the ampule of API C medium provided. Ampule API C was then homogenized and 135 µl was added to each of the 32 wells of the ID32 C-strip. Once all the wells had been filled, the lid was placed on each strip, and they were incubated in a closed container. During the duration of the experiment paper towels dampened with autoclaved distilled water were used to prevent desiccation of the strips. The containers were left at room temperature and photographed 2 wk and 4 wk after the start of the experiment. Two replicates were performed for the wildtype and for each of the 17 *alb* mutants.

### DNA extraction

Whole cell DNA from *E. dermatitidis* wildtype and mutant strains was extracted using the DNeasy PowerSoil Pro Kit (Qiagen) following the manufacturer's protocol. Strains were initially plated on YPD. In total, 51 mutant strains were picked which included the wildtype control, all 17 *alb* mutants, and 33 *hyp* mutants. The *hyp* mutants were picked based on cell morphology data such that the collection of mutants exhibited the greatest diversity of cell type morphologies. Other criteria taken into consideration were the results of the UV and temperature assays. The *hyp* mutants picked represented different growth and inhibition profiles for these phenotypic assays. DNA concentrations were determined using a NanoDrop 2000/2000c Spectrophotometer (Fisher Scientific). The samples were stored at −80 °C for further downstream analysis.

### Genome sequencing

Extracted whole cell DNAs from *E. dermatitidis* strains were shipped for sequencing to the Microbial Genome Sequencing Center (Pittsburgh, USA) or SeqCoast, LLC (Portsmouth, USA) with both vendors using the Illumina platform. The variant caller breseq (current version 0.35.4) was employed by each vendor to align and compare the sequencing data to the *E*. *dermatitidis* UT8656 reference genome (NCBI RefSeq Assembly GCF_000230625.1). *breseq* is a computational pipeline that employs NGS sequence reads in FASTQ format and aligns them to the reference genome sequence files in GenBank, GFF3 or Fasta format (Barrick et al. 2014; Deatherage and Barrick 2014). The resulting dataset provided single nucleotide variants (SNVs) that facilitated identification of the mutations potentially responsible for loss of melanin production and determining the degree of similarity between the mutations observed in the *alb* and *hyp* classes of mutants.

For each sample, a summary statistic file is provided on the number of reads and their alignment rate to the reference genome (Supplementary Fig. 4a). Percentages above >90% are favorable and anything below indicates low quality sequencing data due to poorly constructed DNA fragment libraries. *breseq* utilized 3 types of information to predict mutations: read alignment (RA), missing coverage (MC) and new junction (JC) evidence. For this dataset, RA evidence provided evidence to support mutations resulting in single nucleotide substitutions and deletions that are shorter than the read length. The index.html file obtained from the analysis displayed the “Mutation predictions” page that contained tables listing differences (SNVs) found in the samples and the reference genome. The SNVs were identified based on the RA evidence and the annotation found in the index.html file. The file also provided information regarding the gene and the description based on the reference genome (Supplementary Fig. 4b). For each sample, all the SNVs with RA evidence and annotations were pulled out and compiled in a single file (Supplementary File 1). The SNVs were prioritized based on the type of mutations. Small deletions or substitutions (coding) that shifted the reading frame and nonsense mutations were classified as high priority, non-synonymous missense mutations were classified as medium priority and synonymous SNVs and mutations that resulted in no amino acid change were classified as low priority. The gene description was then taken into account to identify mutations that occurred in genes known to be involved in melanin production in black yeasts. Other genes known to be involved in cell morphology were also given priority. To confirm the geneID description obtained in the index.html files, all genes with mutations were blasted in NCBI BLAST database to confirm their description and also to identify if any new annotations for the hypothetical proteins have been added to the NCBI database for *E. dermatitidis*. The last part of the genome sequencing analysis involved searching for any shared mutations that occurred in all mutants (both *alb* and *hyp*), *alb* mutants only and *hyp* mutants only to see if any significant patterns can be observed between the mutant strains.

### Pks1 structure and representation of mutated *PKS1* sites

To determine if there was a trend that appeared between the conditional *alb* and obligate *alb* mutants, *PKS1* SNVs were obtained from the genome sequences for all 17 *alb* mutants representing the 2 types (conditional and obligate) mutant strains. Domains and location of Pks1 in *E. dermatitidis* were determined using antiSMASH fungal version 7 (Blin et al. 2023) software and the *PKS1* sequences from each *alb* strains and the wildtype control. Based on the position, the *PKS1* sequence from the different *alb* strains aligned to different domains of the reference Pks1.

## Results

### Mutant identification

**Fig. 2.**
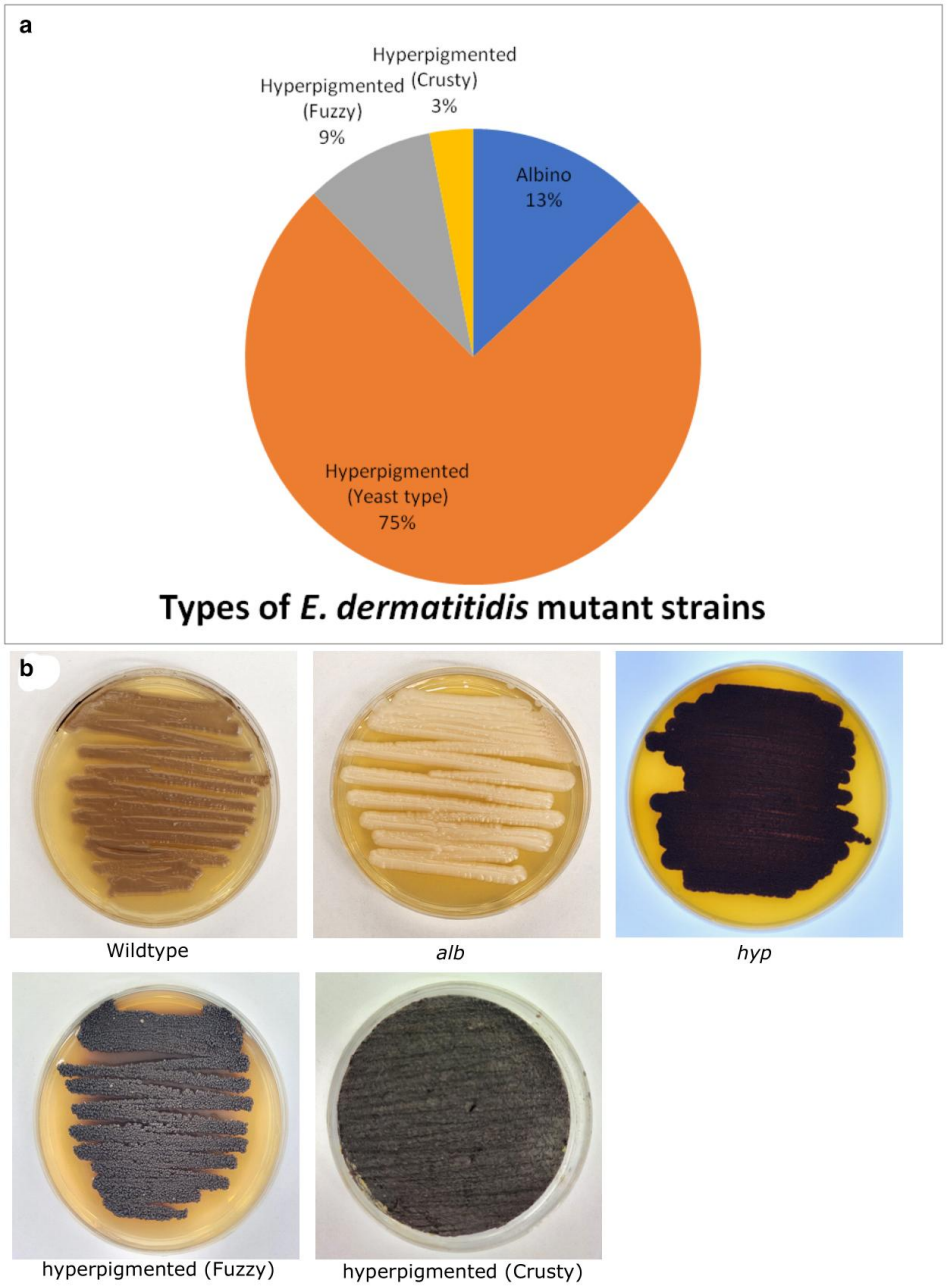
a) Breakdown of the 130 mutants based on phenotypes obtained using random UV mutagenesis on wildtype *E. dermatitidis* reference strain UT8656. b) Images representing the wildtype and mutant *E. dermatitidis* mutant strains obtained in this study: *alb*, *hyp*, hyperpigmented (fuzzy), and hyperpigmented (crusty).

Previous attempts to understand the genetic basis of pigment production in polyextremotolerant fungi have relied upon targeted mutagenesis of specific genes with predicted roles in the production of melanin or carotenoids. A limitation of this approach is its underlying bias toward known genes. We propose that a systematic unbiased screen offers the potential to identify any mutable gene with a role in pigment production. Although *E*. *dermatitidis* is not considered as routinely genetically tractable, the use of genome re-sequencing and variant calling makes it possible to provide some insight into the genetic basis of the recovered mutant phenotypes. Accordingly, UV mutagenesis was employed to recover 130 mutants with pigmentation defects that were selected for further analysis. Out of the 130 mutants that were isolated, 17 were classified as albino (*alb*) mutants and 113 as hyperpigmented (*hyp*) mutants (Fig. 2a). *hyp* mutants were further subdivided into 3 subcategories; (i) those that exist primarily in the yeast form, which made up the vast majority of the *hyp* mutants (97), (ii) hyperpigmented (fuzzy) mutants (12), and (iii) hyperpigmented (crusty) mutants (4) (Fig. 2b).

### Cell and colony morphology

**Fig. 3.**
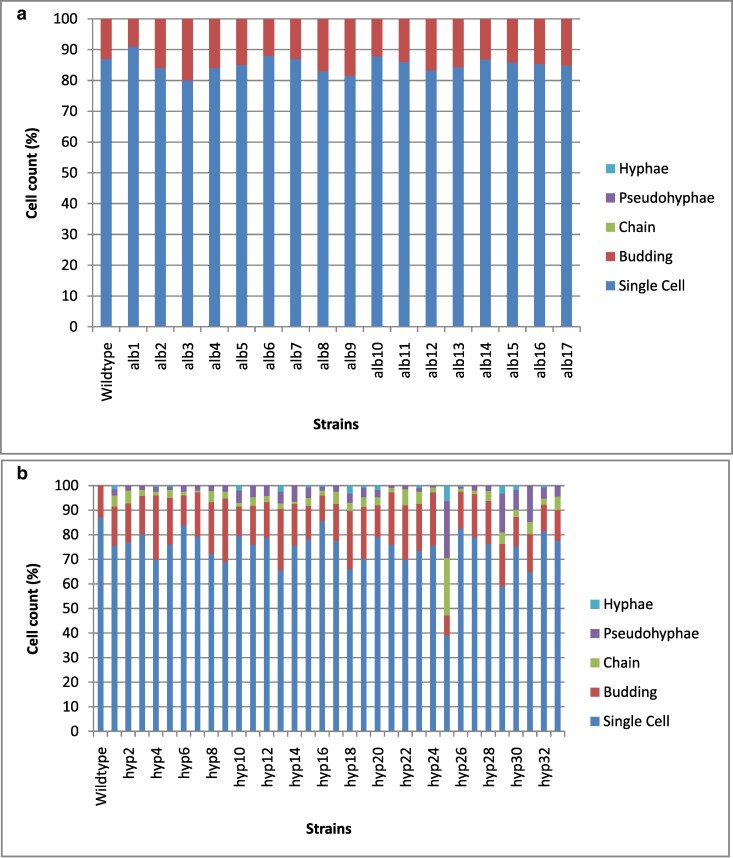
a) Counts of different cell morphologies of *E. dermatitidis alb* mutant strains. b) Counts of different cell morphologies of a subset of *hyp* mutant strains that were selected for genome sequencing (SNV analysis). Colors that define the type of observed cell morphologies as shown on the right. Cell count (%) on *y* axis and strains on *x* axis. Cell count of the remaining *hyp* mutants that were not selected for SNV analysis is shown in Supplementary Fig. 6.

All the *alb* mutants had a yeast-like appearance that included the presence of both budded and unbudded cells (Fig. 3a). The morphology of the *hyp* mutants varied, 50 possessed either unbudded or budded yeast cells, 13 exhibited 3 different cell types (unbudded yeast cells, budded yeast cells, and chains), 24 showed 4 different cell types (unbudded yeast cells, budded yeast cells, chains, and pseudohyphae), and 21 showed all 5 cell types (unbudded yeast cells, budded yeast cells, chains, pseudohyphae, and hyphae) (Fig. 3b) (Supplementary Fig. 5). There were 3 hyperpigmented mutants with 3 cell types that did not contain chains but pseudohyphae instead (unbudded yeast cells, budded yeast cells, and pseudohyphae) (Fig. 3b). Figure 3b only shows cell counts for a subset of *hyp* mutants (strains which were part of the SNV analysis), and Supplementary Fig. 6 shows the cell count morphology for the remaining *hyp* mutants.

### UV resistance and temperature sensitivity

**Fig. 4.**
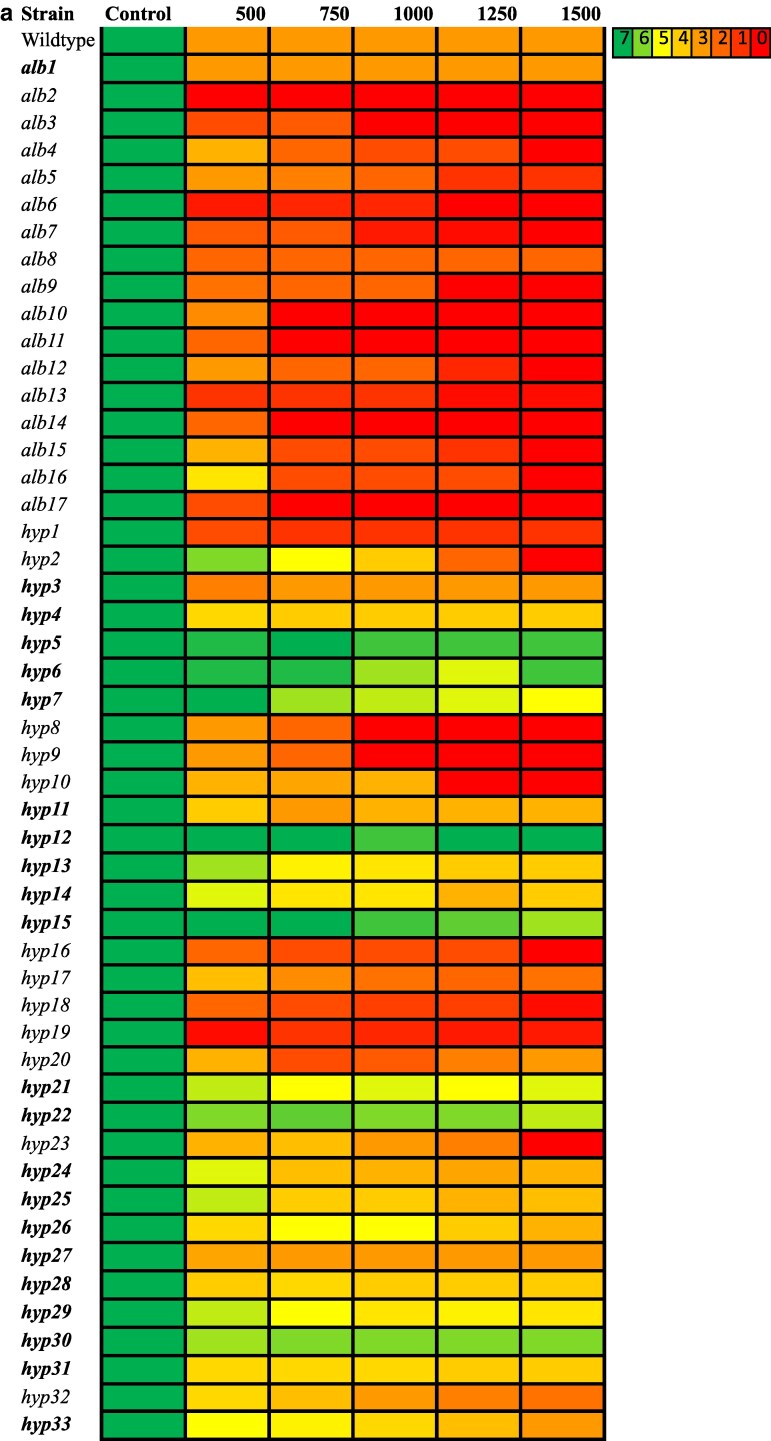

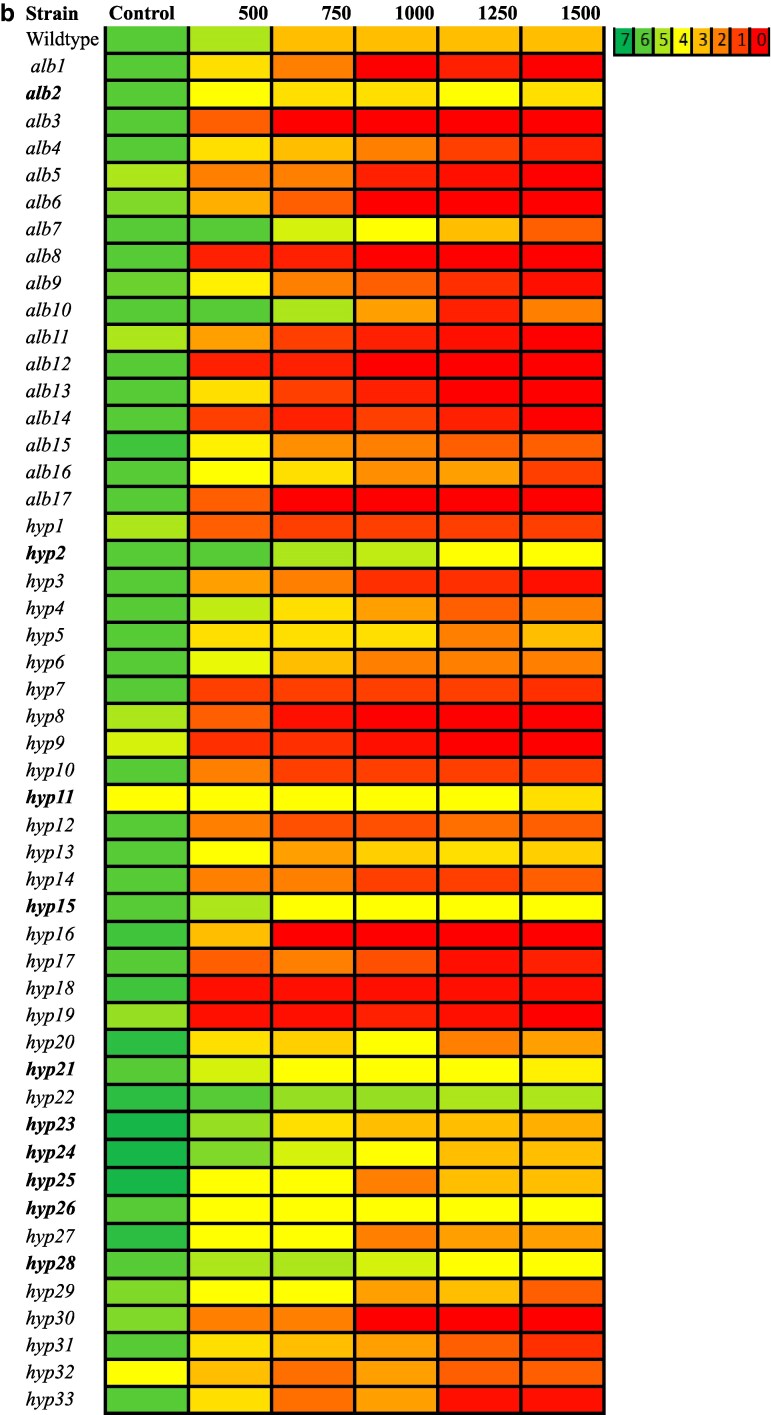
a) Growth observed for *E. dermatitidis* wildtype, *alb*, and subset of *hyp* strains (1:100 dilution) on YPD media at different UV intensities (control [no UV], 500, 750, 1,000, 1,250, and 1,500 energy 1,000 × 100 µW/cm^2^). Colors of the heatmap represent the scores obtained by visualizing the type of growth observed for each spot and comparing them to growth patterns as indicated in Supplementary Fig. 3. Mutant strains that had similar or lower UV intensity at each dose than the wildtype are in bold. Data for remaining *hyp* mutants for 1:100 dilution in Supplemental Figure and data for 1:1, 1:10, and 1:1,000 dilutions for all mutants in Supplementary File 2. b) Growth observed for *E. dermatitidis* wildtype, *alb*, and subset of *hyp* strains (1:100 dilution) on MN media at different UV intensities (control [no UV], 500, 750, 1,000, 1,250, and 1,500 energy 1,000 × 100 µW/cm^2^). Colors of the heatmap represent the scores obtained by visualizing the type of growth observed for each spot and comparing them to growth patterns as indicated in Supplementary Fig. 3. Mutant strains that had similar or better UV resistance compared to the wildtype for each UV dose are in bold. Mutant strains that had similar or better UV resistance compared to the wildtype for each UV dose are in bold. Data for remaining *hyp* mutants for 1:100 dilution are shown in Supplementary Fig. 7b and data for remaining dilutions for all mutants are shown in Supplementary File 2.

Compared to wildtype, the *alb* mutants generally displayed moderate growth reduction at higher UV intensities (Supplementary Fig. 3) when grown on YPD (Fig. 4a) or MN (Fig. 4b) plates. The only exceptions were, *alb1*, which showed similar growth to the wildtype on YPD and *alb2* which showed similar growth to the wildtype on MN. The *hyp* mutants exhibited a range of responses to UV irradiation when incubated on YPD and MN (Supplementary Fig. 7a and b). Whereas some showed dose-dependent sensitivity similar to that of the *alb* mutants, others were indistinguishable from the wildtype. In relation to the media type, the overall trend showed better growth for all mutants on YPD compared to MN during the UV assays.

**Fig. 5.**
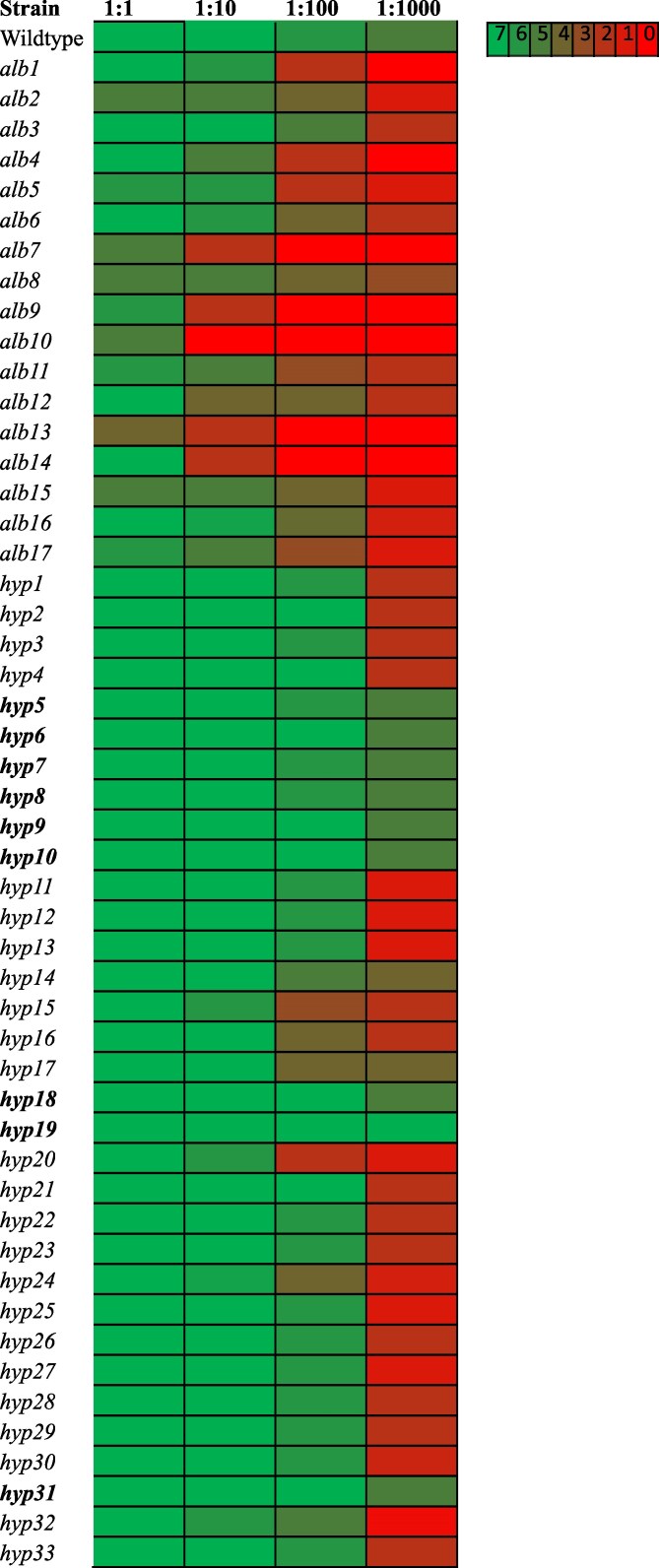
Heatmap representing the growth observed for different dilutions of *E. dermatitidis* wildtype, *alb*, and subset of *hyp* mutant strains growing at 42 °C. Samples were grown on YPD media. Colors of the heatmap represent the scores obtained by visualizing the type of growth observed for each spot and comparing them to growth patterns as indicated in Supplementary Fig. 3. Mutant strains that had similar growth to wildtype at 42 °C are in bold. Data for remaining *hyp* mutants at 42 °C are shown in Supplementary Fig. 8 and data for all mutants at 10 and 28 °C in Supplementary File 2.

Profiling of growth at different temperatures revealed that the *alb* and *hyp* mutants displayed levels of growth comparable to wildtype controls when grown at 10 or 28 °C (Supplementary File 2). At 42 °C, some reduction in growth of the *alb* mutants was observed at all dilutions with complete inhibition at the 1:1,000 dilutions. Conversely, most of the *hyp* mutants were able to grow with little inhibition at 1:1, 1:10, and 1:100 dilutions with some reduction observed only at 1:1,000 dilutions (Fig. 5 and Supplementary Fig. 8).

### Carbon source utilization

**Fig. 6.**
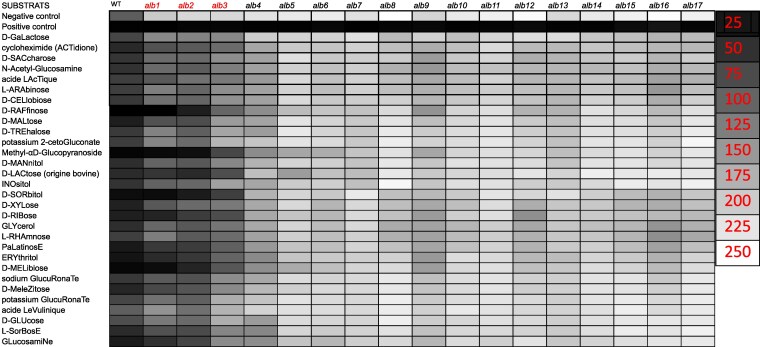
Heatmap representing the growth and melanin production of conditional albinos (*alb1*, *alb2*, *alb3*) compared to the obligate albinos. Intensity values obtained using ImageJ and a cutoff of 100 was used to consider if melanin production was present. Negative control has no substrate and positive control = esculinferric citrate.

Preliminary testing of all 17 *alb* mutants for growth on YPD, YPG, MN, and MEA media yielded the surprising observation that 3 albino mutants (*alb1*, *alb2*, and *alb3*) were able to produce melanin on YPG. To determine in a more systematic manner how alternative carbon sources might impact the phenotypes displayed by the *alb* and *hyp* mutants, ID 32 C-strips were used (Supplementary Fig. 9). The wildtype control was able to grow and produce melanin on all 32 carbon sources, whereas the same 3 *alb* mutants (*alb1*, *alb2*, and *alb3*) were unexpectedly able to produce melanin on multiple carbon sources (Fig. 6). Pixel density obtained using color intensity values less than 100 were used as a cutoff to determine if melanin production was observed. Based on the cutoff, *alb1* was able to reinitiate melanin production on 25 out of 32 carbon sources, *alb2* was able to reinitiate melanin production on 30 out of 32 carbon sources, and *alb3* was able to reinitiate melanin production on 14 out of 32 different carbon sources. All 3 conditional *alb* mutants (*alb1*, *alb2*, and *alb3*) were able to produce melanin on the following 13 carbon sources: D-galactose, cycloheximide (actidione), acide lactique, D-raffinose, methyl-αD-glucopyranoside, D-LACtose (origine bovine), D-sorbitol, D-xylose, D-ribose, glycerol, Palatinose, erythritol, and D-melibiose. Based on the cutoff the remaining 14 albino mutant strains were not able to recover melanin production (Fig. 6).

### Analysis of SNVs

Genomic DNA from all 17 *alb* mutants and 33 selected *hyp* mutants (see the Materials and methods for an explanation of the selection criteria) was prepared, submitted for sequencing, and sequences analyzed for variants as described in the Materials and methods. The number of SNVs varied quite significantly between samples. The *E. dermatitidis* wildtype strain used for these studies (UT86568) possessed 7 synonymous, 4 coding and 13 missense SNVs that were shared by all mutant strains and were not included in the number of unique SNVs found in each mutant strain. The lowest number of SNVs was zero for *hyp19* and *hyp24* and the highest number of SNVs observed was 106 for the *hyp28* mutant (Table 1). Among the albino mutants, the number of SNVs ranged from 48 (*alb15*) to 1 (*alb3*). Out of the 17 *alb* mutants, the top 3 mutants with the highest number SNVs predicted to have high impact were *alb12*, *alb15*, and *alb2* with 33, 32, and 31, respectively. Out of the 33 *hyp* mutants, the top 3 mutants with the highest number of high priority SNVs were *hyp28*, *hyp22*, and *hyp10* with 65, 45, and 43 high priority SNVs, respectively. In all the mutant strains tested, the largest numbers of mutations were missense mutations.

**Table 1.** Total number of SNVs found in the *alb* and *hyp* mutants.

Strains	Total	High impact	Medium impact	Low impact
*alb1*	13	3	5	5
*alb2*	44	5	26	13
*alb3*	16	1	12	3
*alb4*	23	2	20	1
*alb5*	44	2	28	14
*alb6*	35	2	25	8
*alb7*	6	0	5	1
*alb8*	14	0	10	4
*alb9*	12	3	5	4
*alb10*	25	5	17	3
*alb11*	37	4	21	12
*alb12*	43	8	25	11
*alb13*	2	0	2	0
*alb14*	10	2	4	4
*alb15*	48	3	29	16
*alb16*	24	2	15	7
*alb17*	45	8	22	15
*hyp1*	37	5	25	7
*hyp2*	1	1	0	0
*hyp3*	4	0	3	1
*hyp4*	43	9	21	13
*hyp5*	16	4	10	2
*hyp6*	17	3	8	6
*hyp7*	1	1	0	0
*hyp8*	26	10	11	5
*hyp9*	18	3	14	1
*hyp10*	64	8	35	21
*hyp11*	10	1	5	4
*hyp12*	9	1	4	4
*hyp13*	20	3	11	6
*hyp14*	9	2	1	6
*hyp15*	6	4	1	1
*hyp16*	49	9	23	17
*hyp17*	16	5	6	5
*hyp18*	2	0	2	0
*hyp19*	0	0	0	0
*hyp20*	22	3	13	6
*hyp21*	27	6	15	6
*hyp22*	63	12	33	18
*hyp23*	6	1	4	1
*hyp24*	0	0	0	0
*hyp25*	56	11	31	14
*hyp26*	8	2	4	2
*hyp27*	16	4	9	3
*hyp28*	106	12	56	38
*hyp29*	6	1	5	0
*hyp30*	7	2	5	0
*hyp31*	8	2	5	1
*hyp32*	21	4	13	4
*hyp33*	57	8	34	15

Variants classified as high impact include nonsense mutations, mutations affecting start codons, and frameshift mutations. Variants classified as medium impact include non-synonymous missense mutations. Variants classified as low impact include synonymous missense mutations and mutations that result in no amino acid change. The *alb1*, *alb2*, and *alb3* mutants display the conditional albino phenotype.

Strikingly, although the annotation of the *E*. *dermatitidis* genome sequence reveals the presence of genes associated with L-DOPA and pyomelanin melanin production, all 17 of the *alb* mutants possessed high or medium impact mutations in the *PKS1* gene (HMPREF1120_01373) (Table 2). No mutations were recovered in any other annotated melanin biosynthetic genes in these mutants. The a*lb15*, *alb16*, and *alb17* mutants possessed nonsense mutation whereas the rest of the albino strains had insertions, deletions and missense mutation in *PKS1*. None of the hyperpigmented mutants or the wildtype mutant had a mutation in *PKS1*. More generally, the vast majority of the recovered mutations were found in genes that are annotated as hypothetical proteins (Supplementary File 1). Other than *PKS1*, there were only 54 examples of the same gene possessing SNVs in different mutants. Mutations in HMPREF1120_05524 (hypothetical protein) were shared between 16 mutant strains (3 *alb* and 13 *hyp* mutants). Mutations in HMPREF1120_00737 (hypothetical protein) were shared between 3 *alb* and 3 *hyp* mutants. Mutations in HMPREF1120_02419 (hypothetical protein) were shared between 3 *alb* and 3 *hyp* mutants. Mutations in HMPREF1120_04157 (MFS transporter, SP family, sugar:H+ symporter) were shared between 4 *hyp* mutants. Mutation in HMPREF1120_06473 (mitogen-activated protein kinase spm1) was shared between 4 *hyp* mutants. Mutations in HMPREF1120_08863 (AP endonuclease 2) was shared between 2 *hyp* and 1 *alb* mutant. Mutations in HMPREF1120_08462 (hypothetical protein) was shared between 2 *hyp* and 1 *alb* mutant. Mutations in HMPREF1120_07859 (Ca^2+^-transporting ATPase) were shared between 3 *hyp* mutants and mutations in HMPREF1120_06469 (hypothetical protein) were shared between 1 *hyp* and 2 *alb* mutants (Table 3). Multiple other mutations were shared between 2 mutants (Supplementary File 1).

**Table 2.** Mutations observed in the polyketide synthase 1 (*pks1*) gene of the *E. dermatitidis* albino strains.

Strains	Mutation	Impact on coding sequence	*HMPREF1120_03173* (polyketide synthase)
Wildtype	N/A	N/A	N/A
*alb1*	A→C transversion	(AAC→CAC)	N578H
*alb2*	T→C transition	(TTT→TCT)	F764S
*alb3*	T→C transition	(TTC→CTC)	F439L
*alb4*	G→A transition	(CAT→TAT)	H2065Y
*alb5*	A→G transition	(TTT→TCT)	F429S
*alb6*	C→G transversion	(TGG→TCG)	W1252S
*alb7*	A→G transition	(CTC→CCC)	L577P
*alb8*	G→A transition	(CAT→TAT)	H2152Y
*alb9*	A→T transversion	(+52/+408)	1 bp insertion
*alb10*	+G insertion	Coding (4,727/6,084 nt)	1 bp insertion
*alb11*	Δ1 bp deletion	Coding (944/6,202 nt)	1 bp DEL 944
*alb12*	2 bp→TC substitution	Coding (1,068 to 1,069/6,084 nt)	2 bp substitution
*alb13*	Δ15 bp deletion	Coding (2,319 to 2,333/6,202 nt)	15 bp DEL
*alb14*	G→A transition	(CAG→TAG)	Q978 stop
*alb15*	C→T transition	(TGG→TGA)	W198 stop
*alb16*	G→A transition	(CAG→TAG)	Q649 stop
*alb17*	A→T transversion	(TTG→TAG)	L251 stop

The *alb1*, *alb2*, and *alb3* mutants display the conditional albino phenotype.

**Table 3.** Shared mutations between different samples.^a^

Gene	Description	Strains found in
HMPREF1120_04157	MFS transporter, SP family, sugar:H+ symporter	*hyp9*, *hyp12*, *hyp17*, *hyp23*
HMPREF1120_00737	Hypothetical protein	*alb2*, *alb10*, *alb15*, *hyp3*, *hyp10*, *hyp23*
HMPREF1120_02419	Hypothetical protein	*alb2*, *alb5*, *alb12*, *hyp6*, *hyp8*, *hyp15*
HMPREF1120_06473	Mitogen-activated protein kinase spm1	*hyp1*, *hyp4*, *hyp10*
HMPREF1120_05524	Hypothetical protein	*alb9*, *alb12*, *alb17*, *hyp1*, *hyp2*, *hyp4*, *hyp5*, *hyp8*, *hyp14*, *hyp15*, *hyp16*, *hyp26*, *hyp28*, *hyp32*
HMPREF1120_08863	AP endonuclease 2	*alb6*, *hyp33*, *hyp10*, *hyp20*, *hyp25*
HMPREF1120_08462	Hypothetical protein	*alb11*, *hyp22*, *hyp20*
HMPREF1120_07859	Ca^2+^-transporting ATPase	*hyp1*, *hyp4*, *hyp28*
HMPREF1120_06469	Hypothetical protein	*alb13*, *alb14*, *hyp28*

The *alb1*, *alb2*, and *alb3* mutants display the conditional albino phenotype. All other *alb* mutants are obligate albinos.

^a^Table showing shared mutations that are shared within at least 3 mutant strains or more. All shared mutations between 2 mutants are shown in Supplementary File 1.

### Representation of PKS1 mutation sites

**Fig. 7.**
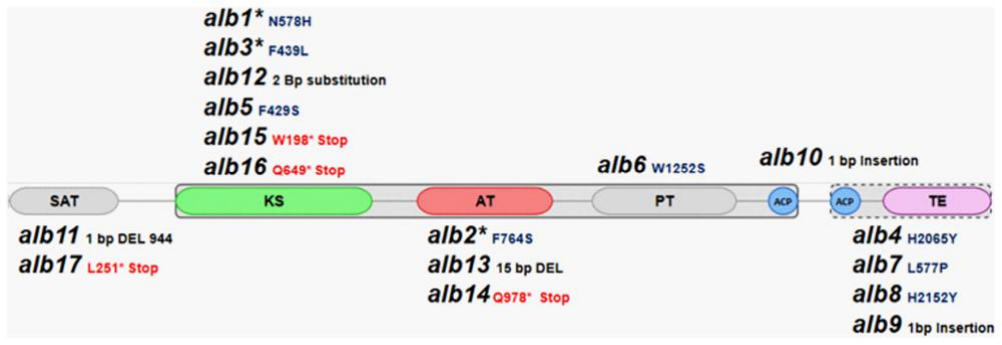
Mutations observed in the polyketide synthase 1 of the *E. dermatitidis* albino strains. The Pks1 domains a schematically represented (starter unit: ACP transacylase [SAT], ketosynthase [KS], acyltransferase [AT], product template [PT], acyl-carrier protein [ACP], and thioesterase [TE]) that are responsible for different aspects of the polyketide synthase pathway in *E. dermatitidis*. *Indicates to conditional albinos. Pks1 amino acid scale with regard to the position of the various domains: SAT: 4 to 241 AA, KS: 368 to 798 AA, AT: 898 to 1,194 AA, PT: 1,282 to 1,598 AA, ACP: 1,669 to 1,733, and 1,805 to 1,870 AA, TE: 1,921 to 2,157 AA.

To assess the possibility that the conditional albino mutants *alb1*, *alb2*, and *alb3* might possess mutations that reside in a specific domain or region of Pks1, the locations of all *alb* mutations were mapped onto the protein domain map of Pks1 (Fig. 7). Conditional albino strains *alb1* and *alb3* possess mutations in the ketoacyl synthase (KS) domain and conditional albino *alb2* has a mutation in the acyltransferase (AT) domain. Of the remaining 14 obligate albinos, 2 strains (*alb11* and *alb17*) had a mutation in the starter-unit acyltransferase (SAT) domain, 4 strains (*alb5*, *alb12*, *alb15*, and *alb16*) harbored a mutation in the ketoacyl synthase (KS) domain, 2 strains (*alb13* and *alb14*) possess a mutation in the acyltransferase (AT) domain, *alb6* has a mutation in the phosphodiesterase (PT) domain, *alb10* possesses a mutation in the acyl-carrier protein (ACP) domain and 4 strains (*alb4*, *alb7*, *alb8*, and *alb9*) exhibit mutations in the thioesterase (TE) domain.

## Discussion

This study represents the first systematic attempt to use unbiased random mutagenesis to investigate the genetic control of melanin production in the model polyextremotolerant fungus *E. dermatitidis*. Previous studies using targeted mutagenesis suggested that 1,8-DHN melanin synthesized via Pks1 is primarily responsible for melanin production. However, subsequent genome annotation showed the existence of additional melanin biosynthetic pathways whose relative contribution to melanin production is not known. Surprisingly, our genetic screen revealed that all recovered albino mutants possess mutations in the *PKS1* gene. However, an even more surprising observation is that pigmentation could be restored to a subset of these albino mutants by changing the carbon source. These results collectively emphasize the importance of 1,8-DHN melanin to growth and stress response in *E. dermatitidis*, while also hinting at surprisingly complex regulatory processes that appear to underlie melanin production.

### *E. dermatitidis* and albino mutants due to mutations in *PKS1*

All *alb* mutants obtained via random mutagenesis shared a mutation in *PKS1* indicating a disruption in the production of 1,8-DHN melanin (Alspaugh et al. 1998; Fujii et al. 2000).The Pks1 enzyme possesses similar structures and domains across the fungi. The type 1 nonreducing Pks is a large complex protein found in fungi has 3 core domains: acyltransferase (AT), acyl-carrier protein (ACP), and ketosynthase (KS) domains (Sabatini et al. 2018; Jia et al. 2021). The ketosynthase (KS) domain is responsible for the elongation of the polyketide backbone by catalyzing repeated decarboxylative condensation, the AT domain is responsible for the selection of the extension unit, and the ACP domain contains a phosphopantetheinyl arm to act as a tether for the growing polyketide and the completed polyketides (Hopwood 1997; Bingle et al. 1999; Li et al. 2010). In addition to these 3 core domains, a thioesterase (TE) domain can also be present which is responsible for either the release of the bound enzyme intermediates or drives the final cyclization reaction hence releasing the final product (Du and Lou 2010). The final product can either be a heptaketide, hexaketide, or pentaketide which is then released by the TE domain (Geyer et al. 2022).

Genome SNV analysis identified variation in the *PKS1* mutations observed in the *E. dermatitidis alb* strains. Mutations were observed in the KS, ACP, AT, and TE domains indicating that all domains are necessary to produce 1,8-DHN melanin and mutations in any of these domains can affect the intermediates necessary to produce 1,8-DHN melanin (Chen et al. 2021). Besides *PKS1* SNVs, the analysis did not identify mutations in any other gene clusters indicating that *PKS1* is the most important polyketide synthase for melanin production in *E. dermatitidis* under routine growing conditions.

In addition to 1,8-DHN melanin, genome annotation of *E. dermatitidis* reveals the presence of pathways that support the production of L-DOPA melanin and pyomelanin (Ito and Wakamatsu 2011; Chen et al. 2014; Solano 2014). In this study, we obtained 3 *alb* mutants (*alb1*, *alb2*, and *alb3*) referred to as conditional because of their ability to produce melanin in response to different carbon sources despite the presence of a mutation in *PKS1*. We speculate that these alternate pathways are responsible for the production of melanin when the 1,8-DHN pathway melanin is disrupted. Expression of genes involved in the L-DOPA pathway and L-Tyrosine degradation pathway has previously been observed in *E. dermatitidis* during skin infection. Under these conditions, expression levels of genes involved in the L-tyrosine degradation pathway were upregulated on skin compared to a negative control whereby *E. dermatitidis* was cultured on prewetted Nylon membrane (Poynter et al. 2016, 2018).

### Carbon utilization

Results from our study suggest that certain carbon sources might be better suited for promoting melanin production. Besides growth, carbon sources have also been shown to influence secondary metabolite production in different fungi (Fang and Zhong 2002; Shih et al. 2007). Genes involved in the sorbicillinoid biosynthesis production via Pks1 in *Ustilaginoidea virens* were shown to be affected by the 12 different carbon sources tested (Meng et al. 2016; Zhang et al. 2023). A recent study on *Aspergillus nidulans* found that DOPA-melanin production was increased when glucose was used as a carbon source (Campanhol et al. 2023). Carbon sources are likely to vary in their ability to produce intermediates involved in the production of different types of melanins in *E. dermatitidis*, which could then account for the differential ability of carbon sources to elicit melanin production in the conditional *alb* mutants. Future experiments will further investigate the expression of the L-DOPA melanin and pyomelanin biosynthetic pathways in these mutants to test this idea.

### UV and temperature resistance

Melanin plays a key role in the adaptation of black yeasts to exposed surfaces. For example, in *Aspergillus niger*, physiological stress caused by UV radiation was also able to enhance the synthesis of melanin as an adaptive response (Singaravelan et al. 2008). Unlike the *alb* mutants, the *hyp* mutants grew much better at higher UV doses indicating the benefit of melanization to counter and mitigate UV stress. Evidence of this comes from a study conducted on *Bipolaris oryzae*, where expression of 1,3,8-trihydroxy-napthalene reductase (*THR1*) gene involved in the production of DHN melanin pathway was also increased when subjected to UV radiation (Kihara et al. 2004).

Melanin can also provide protection from both heat and cold stress. Melanin has the ability to absorb heat energy and dissipate it to shield the cells against heat stress (Paolo et al. 2006). In our study, the *alb* mutant and the *hyp* mutants were able to grow at 10 and 28 °C but at 42 °C, the *hyp* mutants showed a higher growth rate compared to the *alb* mutants. This has been demonstrated in previous studies in *E. dermatitidis* where a *wdpks1*Δ 1 mutation caused a lower survival rate compared to the WT strain that produced melanin (Paolo et al. 2006). In *Cryptococcus*  *neoformans*, cells lacking melanin had a lower survival rate at extreme temperatures than melanized cells (Rosas and Casadevall 1997). The ability of fungi to respond to extreme conditions has led to different adaptations. In fungi the most important signaling pathway stimulated by low and high temperatures is the high-osmolarity glycerol (HOG) signal transduction pathway which is triggered by the sensors in the plasma membrane and leads to the mitogen-activated protein kinase (MAPK) Hog1 via signaling molecules (Winkler et al. 2002; Panadero et al. 2006; Hohmann et al. 2007; Wang et al. 2020). The mutant strains obtained in our study might be utilizing the genes involved in these pathways to mitigate environmental stressors.

### *E. dermatitidis* and hyperpigmented mutant morphology

The genome SNV analysis of the 33 *hyp* mutants failed to reveal evidence for consistent association between a specific gene(s) and the observed phenotypes. The *hyp* mutants obtained in this study were subdivided into yeast-like, fuzzy or crusty morphologies which were attributed to the presence of multicellular pseudohyphae and hyphal filaments. Even within these subgroups there were no strong correlation between genes of known function that contained SNVs and the observed phenotypes. Indeed, the only obvious pattern in all *hyp* mutants was the lack of mutation in *PKS1*, indicating that all *hyp* mutants were able to produce 1,8-DHN melanin similar to the *E. dermatitidis* wildtype strain. Essentially, these results suggest that whereas there may only be 1 (or at least a few) way to generate albino mutants via random mutagenesis, there appears to be a multitude of ways to generate the hyperpigmented phenotype. The broader range of cellular morphologies observed among the *hyp* mutants substantiates this view. The apparent correlation between hyperpigmentation and the formation of hyphae and/or pseudohyphae supports the idea that these are related responses to conditions that reduce growth or cause stress. In that case, it would not be surprising that a greater number of mutations result in hyperpigmentation as this would be a secondary consequence of defects that impact growth or impose stress.

## Conclusion

This study used an unbiased random mutagenesis approach to obtain *alb* and *hyp* mutant strains of the polyextremotolernant black yeast *E. dermatitidis*. The mutants were divided into *alb* and *hyp* mutants based on their phenotypes with regard to their ability to produce melanin. Based on phenotypic assays and melanin recoverability, the *alb* mutants were further subdivided into conditional *alb* that could recover melanin production on different carbon sources and obligate *alb* which had completely lost their ability to recover melanin. Phenotypic characterization based on UV and temperature tolerance revealed that the *hyp* mutants are more stress tolerant than the *alb* mutants. All *alb* mutants possess mutations in *PKS1*, which is involved in the production of 1,8-DHN melanin. Mutations in Pks1 appear to generate an albino phenotype that can be rescued in some mutant backgrounds by substituting dextrose with an alternative carbon source such as galactose as seen in the conditional *alb* mutants. Here galactose could activate signaling pathways that turn on alternative melanin producing pathways or there might be yet unknown functionally redundant components within the main melanin producing pathways that have not yet been deciphered. Future work will incorporate transcriptomics to possibly identify candidate genes/pathways that might be involved in generating conditional albino mutants.

## Supplementary Material

jkaf205_Supplementary_Data

## Data Availability

The authors affirm that all data necessary for confirming the conclusions presented in this manuscript are present within the article, figures, and tables. Strains are available upon request. All DNA sequences have been deposited within the SRA database as accession number PRJNA1233822. Supplemental material available at *G3* online.
