# Supplementary material for: Genetic analysis of pigment production in the fungus Exophiala dermatitidis mutant strains obtained via nontargeted UV mutagenesis
Source: G3 (Bethesda). 2025 Sep 3;15(12):jkaf205. doi: 10.1093/g3journal/jkaf205 (PMC12693494; doi:10.1093/g3journal/jkaf205)
Supplement: jkaf205_Supplementary_Data [file jkaf205_supplementary_data.zip › Supplemental_Figures_G3-2025-406059.docx]

**Supplemental Figures**

**
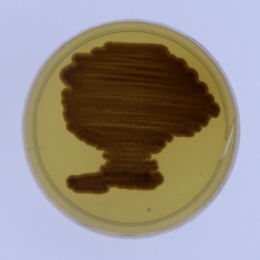
**

**Supplemental Figure 1**: *Exophiala dermatitidis* wildtype strain UT8656 growing on YPD media.


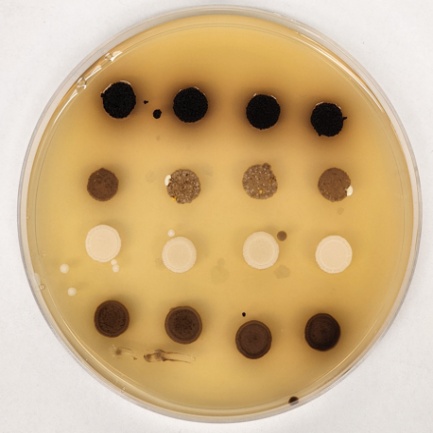

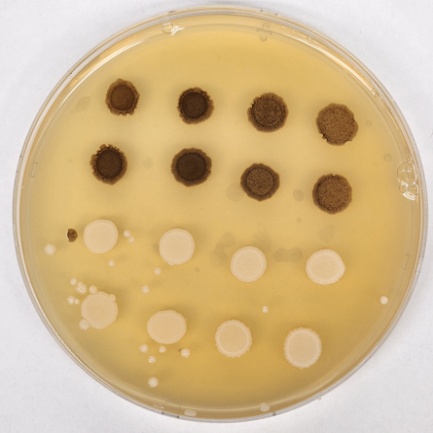

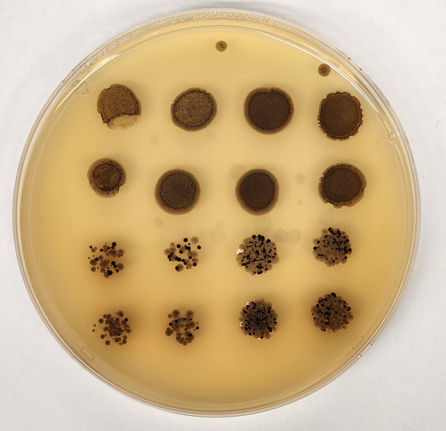


**Supplemental Figure 2:** Example images of spot test plates used to calculate growth and inhibition during UV and temperature assays. Solid spots indicate no inhibition in growth, transparent and scattered spots indicate growth inhibition.

1.
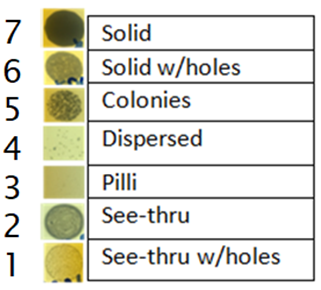
(b)

**Supplemental Figure 3:** Scoring method used to categorize growth and inhibition of *E. dermatitidis* mutant strains during the UV and Temperature Assays. (a) A score of 7 indicates no inhibition and 1 indicates complete inhibition. (b) Image representing the colors associated with the scoring method for heatmaps.

(a)
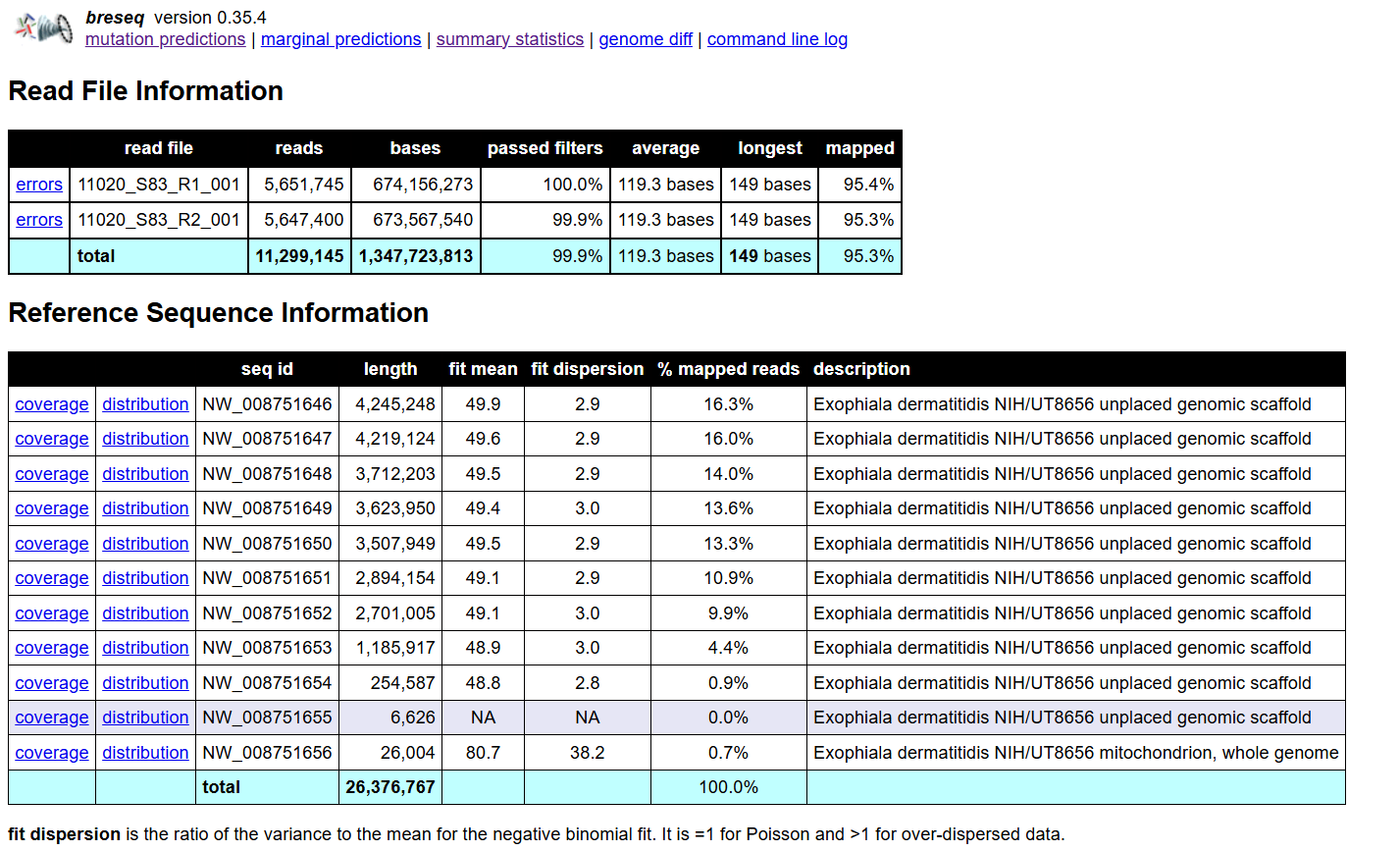


(b)
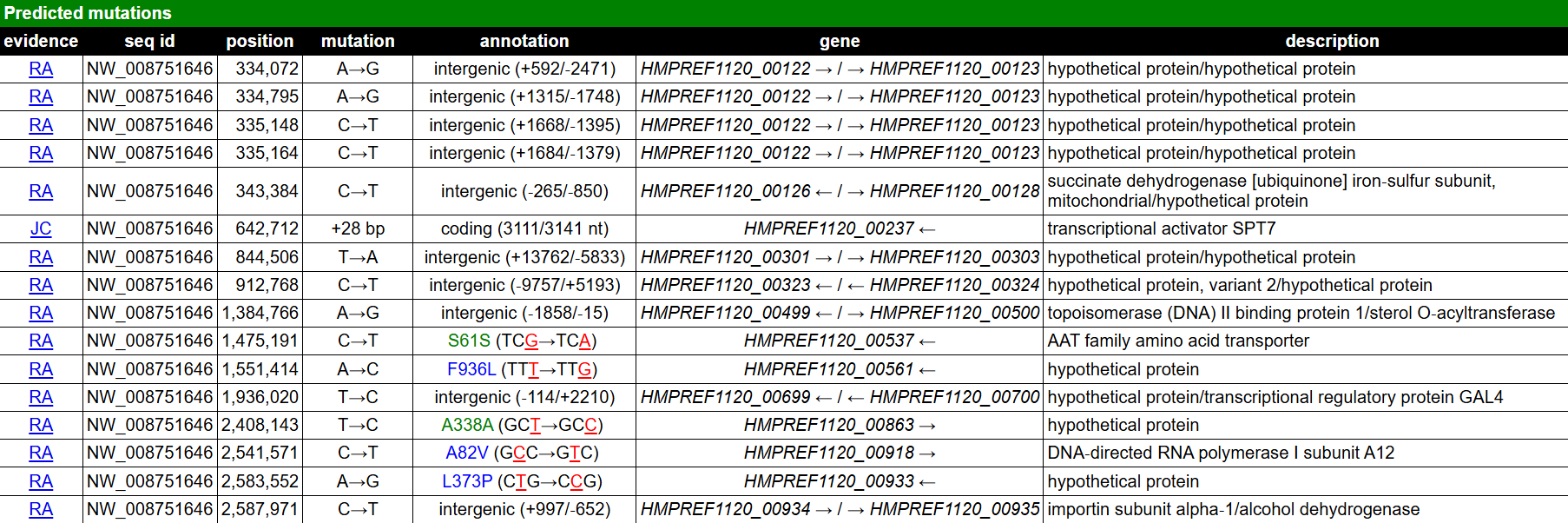


**Supplemental Figure 4:** Example images for one of the sample showing (a) summary statistic file showing the alignment rate and (b) index.html file of the showing the predicted mutations obtained via the breseq computation pipeline showing the evidence, annotation, gene ID and description of the gene.


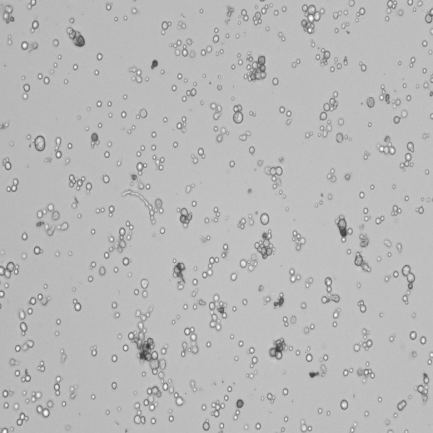

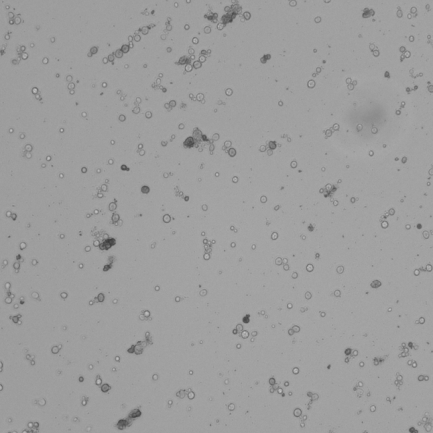

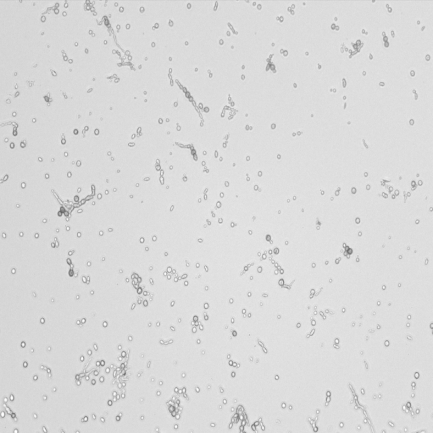


**Supplemental Figure 5:** Images obtained using EVOS M5000 (FL Auto 2 Cell Imaging software) representing the different cell morphology observed in the wildtype and mutant *E. dermatitidis* strains.

**Supplemental Figure 6:** Counts of different cell morphologies of the remaining E*. dermatitidis* *hyp* mutant strains that were not selected for genome sequencing (SNV analysis). Legend indicating the type of cells observed. Cell count (%) on y-axis and strains on x-axis.

| **Sample** | **Control** | **500** | **750** | **1000** | **1250** | **1500** |
| --- | --- | --- | --- | --- | --- | --- |
| Wildtype |  |  |  |  |  |  |
| *hyp34* |  |  |  |  |  |  |
| *hyp35* |  |  |  |  |  |  |
| *hyp36* |  |  |  |  |  |  |
| *hyp37* |  |  |  |  |  |  |
| *hyp38* |  |  |  |  |  |  |
| *hyp39* |  |  |  |  |  |  |
| *hyp40* |  |  |  |  |  |  |
| ***hyp41*** |  |  |  |  |  |  |
| *hyp42* |  |  |  |  |  |  |
| ***hyp43*** |  |  |  |  |  |  |
| *hyp44* |  |  |  |  |  |  |
| *hyp45* |  |  |  |  |  |  |
| *hyp46* |  |  |  |  |  |  |
| ***hyp47*** |  |  |  |  |  |  |
| *hyp48* |  |  |  |  |  |  |
| ***hyp49*** |  |  |  |  |  |  |
| *hyp50* |  |  |  |  |  |  |
| *hyp51* |  |  |  |  |  |  |
| ***hyp52*** |  |  |  |  |  |  |
| *hyp53* |  |  |  |  |  |  |
| *hyp54* |  |  |  |  |  |  |
| ***hyp55*** |  |  |  |  |  |  |
| ***hyp56*** |  |  |  |  |  |  |
| ***hyp57*** |  |  |  |  |  |  |
| *hyp58* |  |  |  |  |  |  |
| ***hyp59*** |  |  |  |  |  |  |
| ***hyp60*** |  |  |  |  |  |  |
| *hyp61* |  |  |  |  |  |  |
| *hyp62* |  |  |  |  |  |  |
| ***hyp63*** |  |  |  |  |  |  |
| *hyp64* |  |  |  |  |  |  |
| ***hyp65*** |  |  |  |  |  |  |
| ***hyp66*** |  |  |  |  |  |  |
| *hyp67* |  |  |  |  |  |  |
| ***hyp68*** |  |  |  |  |  |  |
| ***hyp69*** |  |  |  |  |  |  |
| *hyp70* |  |  |  |  |  |  |
| *hyp71* |  |  |  |  |  |  |
| *hyp72* |  |  |  |  |  |  |
| *hyp73* |  |  |  |  |  |  |
| *hyp74* |  |  |  |  |  |  |
| *hyp75* |  |  |  |  |  |  |
| *hyp76* |  |  |  |  |  |  |
| *hyp77* |  |  |  |  |  |  |
| *hyp78* |  |  |  |  |  |  |
| ***hyp79*** |  |  |  |  |  |  |
| ***hyp80*** |  |  |  |  |  |  |
| *hyp81* |  |  |  |  |  |  |
| *hyp82* |  |  |  |  |  |  |
| ***hyp83*** |  |  |  |  |  |  |
| *hyp84* |  |  |  |  |  |  |
| ***hyp85*** |  |  |  |  |  |  |
| ***hyp86*** |  |  |  |  |  |  |
| ***hyp87*** |  |  |  |  |  |  |
| *hyp88* |  |  |  |  |  |  |
| *hyp89* |  |  |  |  |  |  |
| ***hyp90*** |  |  |  |  |  |  |
| ***hyp91*** |  |  |  |  |  |  |
| *hyp92* |  |  |  |  |  |  |
| *hyp93* |  |  |  |  |  |  |
| *hyp94* |  |  |  |  |  |  |
| *hyp95* |  |  |  |  |  |  |
| ***hyp96*** |  |  |  |  |  |  |
| *hyp97* |  |  |  |  |  |  |
| *hyp98* |  |  |  |  |  |  |
| *hyp99* |  |  |  |  |  |  |
| ***hyp100*** |  |  |  |  |  |  |
| *hyp101* |  |  |  |  |  |  |
| *hyp102* |  |  |  |  |  |  |
| ***hyp103*** |  |  |  |  |  |  |
| *hyp104* |  |  |  |  |  |  |
| *hyp105* |  |  |  |  |  |  |
| *hyp106* |  |  |  |  |  |  |
| *hyp107* |  |  |  |  |  |  |
| ***hyp108*** |  |  |  |  |  |  |
| *hyp109* |  |  |  |  |  |  |
| *hyp110* |  |  |  |  |  |  |
| ***hyp111*** |  |  |  |  |  |  |
| *hyp112* |  |  |  |  |  |  |
| ***hyp113*** |  |  |  |  |  |  |

**Supplemental Figure 7a**: Growth observed for the remaining *E. dermatitidis* *hyp* strains (1:100 dilution) on YPD media at different UV intensities (control (no UV), 500, 750, 1000, 1250 and 1500 Energy 1000 x 100µW/cm^2^). Colors of the heatmap represent the scores obtained by visualizing the type of growth observed for each spot and comparing them to growth patterns as indicated in Supplemental Figure 3. Mutant strains that had similar or lower UV intensity than the wildtype are in bold. Data for 1:1, 1:10 and 1:1000 dilutions for all mutants in Supplemental File 2, Worksheet 1.

| **Sample** | **Control** | **500** | **750** | **1000** | **1250** | **1500** |
| --- | --- | --- | --- | --- | --- | --- |
| Wildtype |  |  |  |  |  |  |
| *hyp34* |  |  |  |  |  |  |
| *hyp35* |  |  |  |  |  |  |
| *hyp36* |  |  |  |  |  |  |
| ***hyp37*** |  |  |  |  |  |  |
| *hyp38* |  |  |  |  |  |  |
| *hyp39* |  |  |  |  |  |  |
| *hyp40* |  |  |  |  |  |  |
| ***hyp41*** |  |  |  |  |  |  |
| *hyp42* |  |  |  |  |  |  |
| *hyp43* |  |  |  |  |  |  |
| *hyp44* |  |  |  |  |  |  |
| *hyp45* |  |  |  |  |  |  |
| ***hyp46*** |  |  |  |  |  |  |
| ***hyp47*** |  |  |  |  |  |  |
| *hyp48* |  |  |  |  |  |  |
| ***hyp49*** |  |  |  |  |  |  |
| *hyp50* |  |  |  |  |  |  |
| *hyp51* |  |  |  |  |  |  |
| *hyp52* |  |  |  |  |  |  |
| *hyp53* |  |  |  |  |  |  |
| *hyp54* |  |  |  |  |  |  |
| ***hyp55*** |  |  |  |  |  |  |
| ***hyp56*** |  |  |  |  |  |  |
| *hyp57* |  |  |  |  |  |  |
| ***hyp58*** |  |  |  |  |  |  |
| *hyp59* |  |  |  |  |  |  |
| *hyp60* |  |  |  |  |  |  |
| *hyp61* |  |  |  |  |  |  |
| *hyp62* |  |  |  |  |  |  |
| *hyp63* |  |  |  |  |  |  |
| *hyp64* |  |  |  |  |  |  |
| *hyp65* |  |  |  |  |  |  |
| ***hyp66*** |  |  |  |  |  |  |
| *hyp67* |  |  |  |  |  |  |
| *hyp68* |  |  |  |  |  |  |
| ***hyp69*** |  |  |  |  |  |  |
| *hyp70* |  |  |  |  |  |  |
| *hyp71* |  |  |  |  |  |  |
| *hyp72* |  |  |  |  |  |  |
| *hyp73* |  |  |  |  |  |  |
| *hyp74* |  |  |  |  |  |  |
| *hyp75* |  |  |  |  |  |  |
| *hyp76* |  |  |  |  |  |  |
| *hyp77* |  |  |  |  |  |  |
| *hyp78* |  |  |  |  |  |  |
| *hyp79* |  |  |  |  |  |  |
| *hyp80* |  |  |  |  |  |  |
| ***hyp81*** |  |  |  |  |  |  |
| *hyp82* |  |  |  |  |  |  |
| ***hyp83*** |  |  |  |  |  |  |
| ***hyp84*** |  |  |  |  |  |  |
| *hyp85* |  |  |  |  |  |  |
| *hyp86* |  |  |  |  |  |  |
| ***hyp87*** |  |  |  |  |  |  |
| *hyp88* |  |  |  |  |  |  |
| ***hyp89*** |  |  |  |  |  |  |
| *hyp90* |  |  |  |  |  |  |
| *hyp91* |  |  |  |  |  |  |
| *hyp92* |  |  |  |  |  |  |
| *hyp93* |  |  |  |  |  |  |
| *hyp94* |  |  |  |  |  |  |
| ***hyp95*** |  |  |  |  |  |  |
| ***hyp96*** |  |  |  |  |  |  |
| *hyp97* |  |  |  |  |  |  |
| *hyp98* |  |  |  |  |  |  |
| *hyp99* |  |  |  |  |  |  |
| *hyp100* |  |  |  |  |  |  |
| *hyp101* |  |  |  |  |  |  |
| ***hyp102*** |  |  |  |  |  |  |
| *hyp103* |  |  |  |  |  |  |
| *hyp104* |  |  |  |  |  |  |
| ***hyp105*** |  |  |  |  |  |  |
| *hyp106* |  |  |  |  |  |  |
| *hyp107* |  |  |  |  |  |  |
| *hyp108* |  |  |  |  |  |  |
| *hyp109* |  |  |  |  |  |  |
| *hyp110* |  |  |  |  |  |  |
| *hyp111* |  |  |  |  |  |  |
| *hyp112* |  |  |  |  |  |  |
| *hyp113* |  |  |  |  |  |  |

**Supplemental Figure 7b**: Growth observed for the remaining *E. dermatitidis* *hyp* strains (1:100 dilution) on MN media at different UV intensities (control (no UV), 500, 750, 1000, 1250 and 1500 Energy 1000 x 100µW/cm^2^). Colors of the heatmap represent the scores obtained by visualizing the type of growth observed for each spot and comparing them to growth patterns as indicated in Supplemental Figure 3. Mutant strains that had similar or lower UV intensity than the wildtype are in bold. Data for 1:1, 1:10 and 1:1000 dilutions for all mutants in Supplemental File 2, worksheet 2.

| **Sample** | **1:1** | **1:10** | **1:100** | **1:1000** |
| --- | --- | --- | --- | --- |
| Wildtype |  |  |  |  |
| *hyp34* |  |  |  |  |
| ***hyp35*** |  |  |  |  |
| *hyp36* |  |  |  |  |
| *hyp37* |  |  |  |  |
| *hyp38* |  |  |  |  |
| *hyp39* |  |  |  |  |
| *hyp40* |  |  |  |  |
| *hyp41* |  |  |  |  |
| *hyp42* |  |  |  |  |
| *hyp43* |  |  |  |  |
| *hyp44* |  |  |  |  |
| *hyp45* |  |  |  |  |
| *hyp46* |  |  |  |  |
| *hyp47* |  |  |  |  |
| ***hyp48*** |  |  |  |  |
| ***hyp49*** |  |  |  |  |
| *hyp50* |  |  |  |  |
| ***hyp51*** |  |  |  |  |
| ***hyp52*** |  |  |  |  |
| ***hyp53*** |  |  |  |  |
| *hyp54* |  |  |  |  |
| *hyp55* |  |  |  |  |
| *hyp56* |  |  |  |  |
| *hyp57* |  |  |  |  |
| *hyp58* |  |  |  |  |
| *hyp59* |  |  |  |  |
| ***hyp60*** |  |  |  |  |
| ***hyp61*** |  |  |  |  |
| *hyp62* |  |  |  |  |
| ***hyp63*** |  |  |  |  |
| ***hyp64*** |  |  |  |  |
| ***hyp65*** |  |  |  |  |
| *hyp66* |  |  |  |  |
| *hyp67* |  |  |  |  |
| *hyp68* |  |  |  |  |
| ***hyp69*** |  |  |  |  |
| ***hyp70*** |  |  |  |  |
| *hyp71* |  |  |  |  |
| *hyp72* |  |  |  |  |
| *hyp73* |  |  |  |  |
| *hyp74* |  |  |  |  |
| *hyp75* |  |  |  |  |
| *hyp76* |  |  |  |  |
| ***hyp77*** |  |  |  |  |
| *hyp78* |  |  |  |  |
| *hyp79* |  |  |  |  |
| ***hyp80*** |  |  |  |  |
| *hyp81* |  |  |  |  |
| *hyp82* |  |  |  |  |
| *hyp83* |  |  |  |  |
| *hyp84* |  |  |  |  |
| ***hyp85*** |  |  |  |  |
| *hyp86* |  |  |  |  |
| *hyp87* |  |  |  |  |
| ***hyp88*** |  |  |  |  |
| *hyp89* |  |  |  |  |
| *hyp90* |  |  |  |  |
| *hyp91* |  |  |  |  |
| *hyp92* |  |  |  |  |
| *hyp93* |  |  |  |  |
| *hyp94* |  |  |  |  |
| *hyp95* |  |  |  |  |
| *hyp96* |  |  |  |  |
| *hyp97* |  |  |  |  |
| *hyp98* |  |  |  |  |
| *hyp99* |  |  |  |  |
| *hyp100* |  |  |  |  |
| *hyp101* |  |  |  |  |
| *hyp102* |  |  |  |  |
| ***hyp103*** |  |  |  |  |
| *hyp104* |  |  |  |  |
| *hyp105* |  |  |  |  |
| *hyp106* |  |  |  |  |
| *hyp107* |  |  |  |  |
| *hyp108* |  |  |  |  |
| *hyp109* |  |  |  |  |
| *hyp110* |  |  |  |  |
| *hyp111* |  |  |  |  |
| *hyp112* |  |  |  |  |
| *hyp113* |  |  |  |  |

**Supplemental Figure 8**: Heatmap representing the growth observed for different dilutions of remaining *hyp* mutant strains growing at 42°C. Samples were grown on YPD media. Colors of the heatmap represent the scores obtained by visualizing the type of growth observed for each spot and comparing them to growth patterns as indicated in Supplemental Figure 3. Mutant strains that had similar growth to wildtype at 42°C are in bold. Data for 10°C and 28°C for all mutants in Supplemental File 2, worksheet 3.

(a)

(b)

(c)
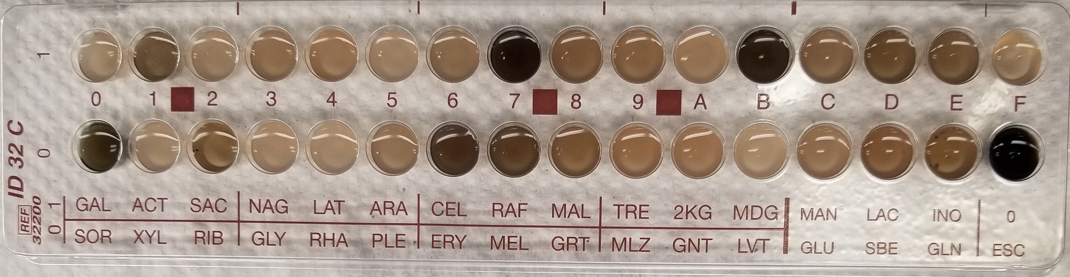


(d)
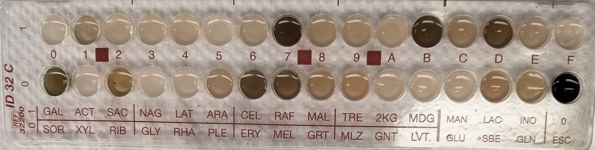


(e)
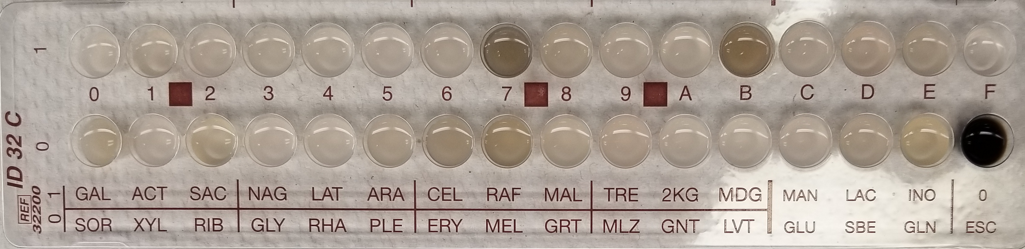


(f)
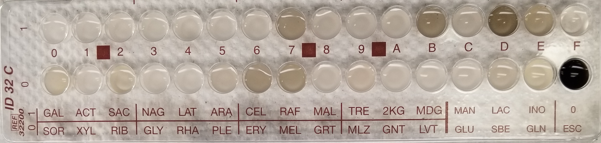


(g)
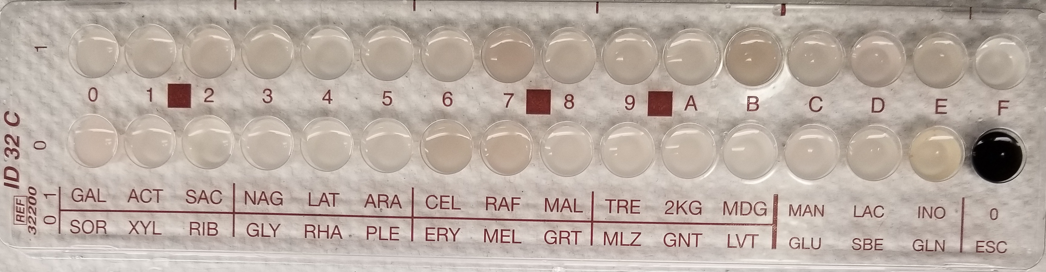


(h)
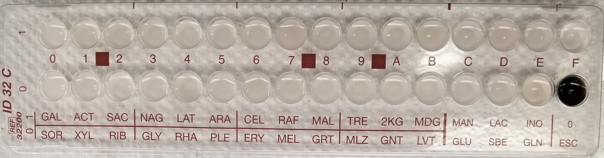


(i)
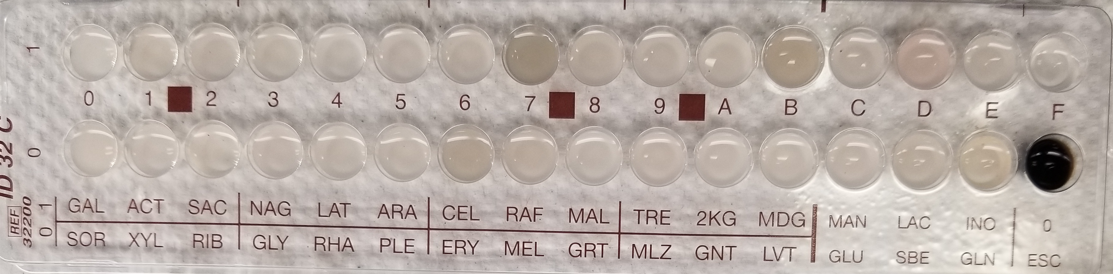


(j)
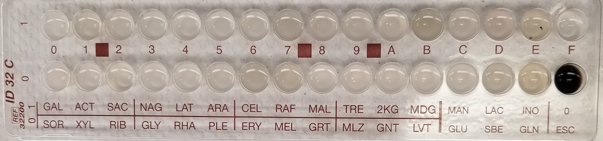


(k)
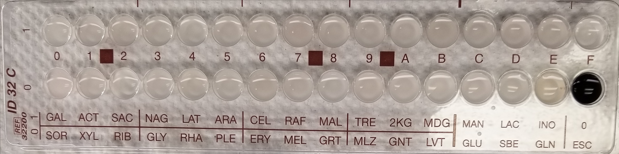


(l)
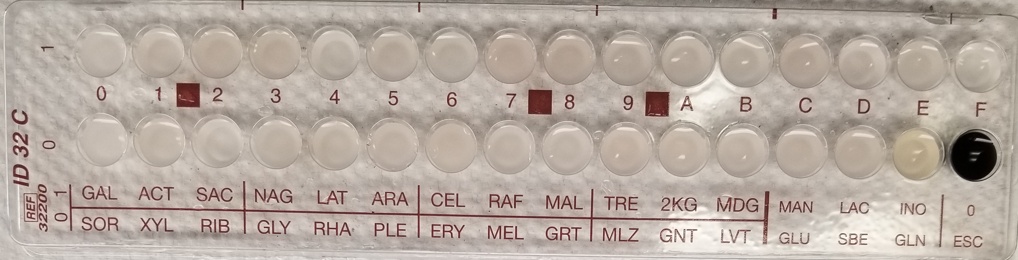


(m)
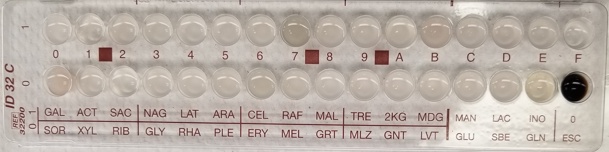


(n)
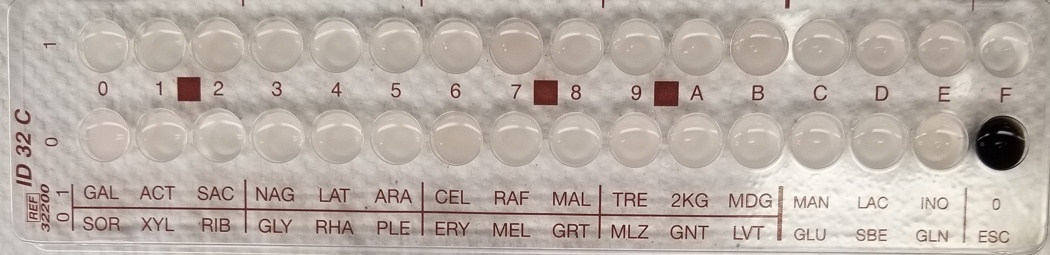


(o)
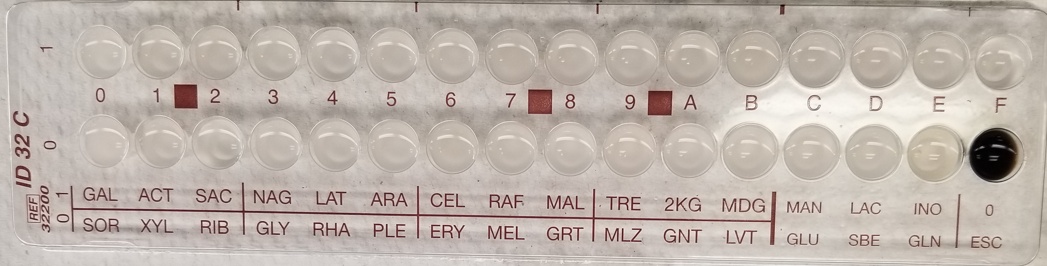


(p)
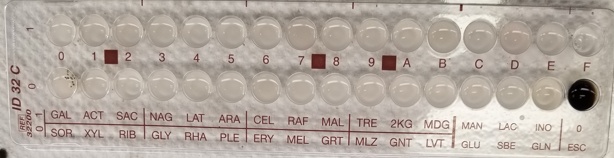


(q)
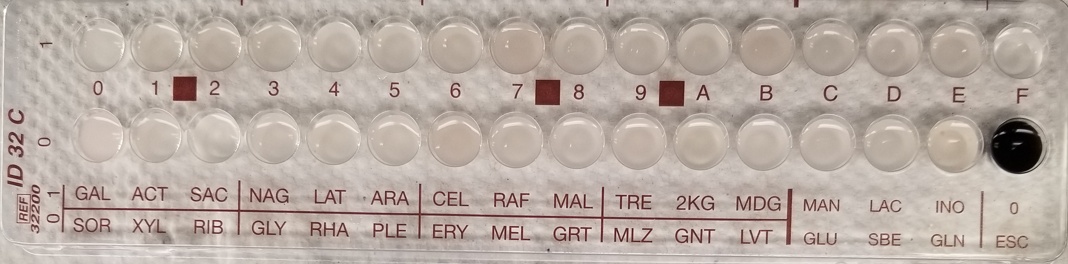


(r)
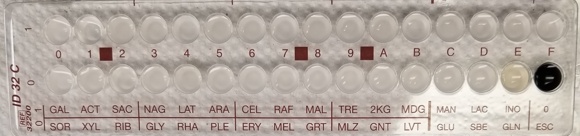


**Supplemental Figure 9:** Images representing the growth of *E. dermatitidis* (a) wildtype, (b) *alb1*, (c) *alb2*, (d) *alb3,* (e) *alb4,* (f) *alb5,* (g) *alb6,* (h) *alb7,* (i) *alb8,* (j) *alb9,* (k) *alb10,* (l) *alb11,* (m) *alb12,* (n) *alb13,* (o) *alb13,* (p) *alb15,* (q) *alb16* and, (r) *alb17* on ID 32-C Strips. *Alb1, alb2 and alb3* are conditional albinos and the rest are obligate albinos. Negative control (1.F) has no substrate and positive control (0.F) includes Esculinferric citrate.
